# Improving Integration of Women's Health Considerations in Psychiatric Inpatient Care: A Quality Improvement Project on Menstrual, Contraceptive, Perinatal, and Menopausal Health Across the Royal Edinburgh Hospital and Associated Services

**DOI:** 10.1192/bjo.2026.11463

**Published:** 2026-06-30

**Authors:** Mary Prempeh, Lina Ling, Rowan Ah-See, Kate Womersley, Katie Marwick

**Affiliations:** 1 University of Edinburgh, Edinburgh, United Kingdom; 2 NHS Lothian, Edinburgh, United Kingdom

## Abstract

**Aims:**

Mental health concerns related to menstruation, the perinatal period, and perimenopause are under-researched healthcare priorities, as identified by the Royal College of Obstetrics and Gynaecology. The links between reproductive life events and psychiatric symptoms are overlooked, particularly in people with chronic mental illnesses.

We aimed to assess whether female reproductive health is being routinely appraised by clinicians caring for inpatients in the Royal Edinburgh Hospital and Associated Services (REAS). Our secondary aim was to identify areas for improvement and consider changes to aid clinician awareness, documentation, and onward referrals.

**Methods:**

We conducted an evaluation to review clerking, ward round, and progress notes using Lothian TrakCare electronic health records. Ten female patients from each of the eight REAS wards (CAMHS, two General Adult Psychiatry wards, Forensics, Mother and Baby Unit, Eating Disorder Unit, Rehabilitation Wards, Old Age Psychiatry) admitted before 31/07/25, were retrospectively included (N=80). Keyword searches were performed for the terms: “menstruation”, “contraception”, “perinatal issues” and “pregnancy status”, “menopause” and “HRT” (Hormone Replacement Therapy); and a score was assigned (1 mention; 0 no mention). Free text from electronic health records was captured and underwent further thematic analysis.

**Results:**

Overall, the documentation of female reproductive health factors was uncommon. Only 20% (N=4/20) of General Adult Psychiatry patients were asked about menstruation and contraception, compared to 70% (N=7/10) in Rehabilitation wards. Issues tended to be raised by patients and not proactively solicited. Conversations regarding menstruation were documented in 100% (N=10/10) and 80% (N=8/10) of Eating Disorder Unit and CAMHS unit notes respectively. This was due to targeted questioning about amenorrhoea and low BMI. Menopausal and HRT documentation was minimal with 17% (N=6/36) and 8% (N=3/36) respectively (inclusion criterion: age ≥ 45).

**Conclusion:**

This service evaluation found minimal documentation of female reproductive health across psychiatric inpatient units. Notable areas of increased documentation include the Rehabilitation wards, Eating Disorder Unit, and CAMHS unit. Our next steps are to engage service users on how they prefer to be asked about reproductive health; to survey relevant clinicians on reproductive health knowledge and confidence; and to implement educational interventions to improve clinician practice.

**Disclosure:** This project was previously submitted as an abstract for presentation at *PsyQIatry: Ideas, Innovation, and Impact in Psychiatry*, March 2026.

*Vikki Argent, Ariana Axiaq, Jo Brown, Michelle Cooper, Flora Flinn, Jenny Green, Katie Halton, Francesca Moss-Lawton, Shani Ross, Penny Shutt, Rob Waller.

